# Repositioning microbial biotechnology against COVID‐19: the case of microbial production of flavonoids

**DOI:** 10.1111/1751-7915.13675

**Published:** 2020-10-13

**Authors:** Tobias Goris, Álvaro Pérez‐Valero, Igor Martínez, Dong Yi, Luis Fernández‐Calleja, David San León, Uwe T. Bornscheuer, Patricia Magadán‐Corpas, Felipe Lombó, Juan Nogales

**Affiliations:** ^1^ Department of Molecular Toxicology, Research Group Intestinal Microbiology German Institute of Human Nutrition Potsdam‐Rehbruecke Arthur‐Scheunert‐Allee 114‐116 Nuthetal Brandenburg 14558 Germany; ^2^ Research Unit “Biotechnology in Nutraceuticals and Bioactive Compounds‐BIONUC” Departamento de Biología Funcional, Área de Microbiología Universidad de Oviedo Oviedo Spain; ^3^ Instituto Universitario de Oncología del Principado de Asturias Oviedo Spain; ^4^ Instituto de Investigación Sanitaria del Principado de Asturias Oviedo Spain; ^5^ Department of Systems Biology Centro Nacional de Biotecnología CSIC Madrid Spain; ^6^ Department of Biotechnology & Enzyme Catalysis Institute of Biochemistry University Greifswald Felix‐Hausdorff‐Str. 4 Greifswald D‐17487 Germany; ^7^ Interdisciplinary Platform for Sustainable Plastics towards a Circular Economy‐Spanish National Research Council (SusPlast‐CSIC) Madrid Spain

## Abstract

Coronavirus‐related disease 2019 (COVID‐19) became a pandemic in February 2020, and worldwide researchers try to tackle the disease with approved drugs of all kinds, or to develop novel compounds inhibiting viral spreading. Flavonoids, already investigated as antivirals in general, also might bear activities specific for the viral agent causing COVID‐19, SARS‐CoV‐2. Microbial biotechnology and especially synthetic biology may help to produce flavonoids, which are exclusive plant secondary metabolites, at a larger scale or indeed to find novel pharmaceutically active flavonoids. Here, we review the state of the art in (i) antiviral activity of flavonoids specific for coronaviruses and (ii) results derived from computational studies, mostly docking studies mainly inhibiting specific coronaviral proteins such as the 3CL (main) protease, the spike protein or the RNA‐dependent RNA polymerase. In the end, we strive towards a synthetic biology pipeline making the fast and tailored production of valuable antiviral flavonoids possible by applying the last concepts of division of labour through co‐cultivation/microbial community approaches to the DBTL (Design, Build, Test, Learn) principle.

## Graphical abstract

Coronavirus‐related disease 2019 (COVID‐19) became a pandemic in February 2020 and worldwide researchers try to tackle the disease with approved drugs of all kinds, or to develop novel compounds inhibiting viral spreading. Flavonoids, already investigated as antivirals in general, also might bear activities specific for the viral agent causing COVID‐19, SARS‐CoV‐2. Microbial biotechnology and especially synthetic biology may help to produce novel flavonoids with antiviral activity.

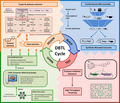

## Introduction

The infectious coronavirus disease 2019 (COVID‐19) caused by the severe acute respiratory syndrome coronavirus 2 (SARS‐CoV‐2) has suddenly become a devastating pandemic that is responsible for a global crisis likely unparalleled since the Second World War (https://www.who.int/emergencies/diseases/novel‐coronavirus‐2019/situation‐reports). While humanity pretentiously assumed such pandemics as evens of the past, COVID‐19 has abruptly brought us back to reality while showing how far we are from being immune to such novel viruses.

The appearance of SARS‐CoV‐2 has forced a variety of scientists worldwide, including immunologists, epidemiologists, mathematicians, physicists and engineers, to change their primary research focus these days with the aim of delivering solutions for the COVID‐19 pandemic. Microbial biotechnologists should be no different. Community‐driven short‐term response to SARS‐CoV‐2 has been capitalized by huge efforts aimed at the development of a large variety of potential vaccines (https://www.who.int/publications/m/item/draft‐landscape‐of‐covid‐19‐candidate‐vaccines), diagnostic tests (Cheng *et al*., 2020), screening of available chemical libraries and/or the repurposing of already approved drugs against SARS‐CoV‐2 (Martinez, 2020). The list of both individual and governmental initiatives grows daily and they represent the first, and so far, unique defence line against the pandemic. However, the urgent need to provide rapid, efficient and cost‐effective solutions against COVID‐19 cannot hide the fact that current chemical structural diversity available for screening is still limited. Therefore, as novel coronaviruses might cross the species barrier leaping from their natural reservoirs to human hosts (Latinne *et al*., 2020), we need an expanded drugs arsenal that should be generated in the medium or long term to fight future coronavirus‐related diseases to come.

Microbial biotechnology is in good shape to lead this second wave of efforts against COVID‐19 and related diseases. Recent advances in systems and synthetic biology have endorsed the field of new and unprecedented tools which, when combined in iterative pipelines, result in powerful approaches to increase chemical diversity and industrial production of bioactive compounds, such as antivirals. For instance, iterative‐learning cycles such as design–build–test–learn (DBTL) cycles have been developed linking the different phases of metabolic engineering fuelled by artificial intelligence (AI), robotic platforms and synthetic biology. These loop platforms not only accelerate optimization processes but also allow a targeted exploration of new chemical space towards multiple applications including drug discovery (HamediRad *et al*., 2019; Wilkinson *et al*., 2020).

The technology and workflow created for accelerating industrial bioprocesses could also help in fighting emergent diseases such as COVID‐19. Therefore, ongoing microbial biotechnology efforts and available resources can be reallocated in a straightforward manner to provide novel antimicrobial drugs, such as those needed for treating coronavirus infections.

We envision, as a paradigmatic example of this repositioning, the biotechnological production of flavonoids. Flavonoids are among the most numerous and widely distributed families of bioactive compounds in plants, with several thousands of representatives (Kumar and Pandey, 2013). Chemically, flavonoids are characterized by a 15‐carbon skeleton (structured as C6‐C3‐C6) with two phenyl aromatic rings (A and B) plus an aromatic heterocyclic ring (C ring), all modified with one or more residual groups, such as hydroxy‐ (glycosylated or not) or methoxy‐ (Kumar and Pandey, 2013; Panche *et al*., 2016). Interestingly, flavonoid biosynthesis follows a radial expansion, thus promoting a huge universe of chemical structures in which the theoretical number by far exceeds the known structures. Flavonoids are split into several subclasses based on the level of substitution and the C‐ring structure: chalcones (such as phloretin); flavanones (such as hesperetin); flavones (such as luteolin); flavonols (such as quercetin); flavan‐3‐ols (such as epigallocatechin); isoflavones (such as genistein); and anthocyanins (such as malvidin). One of the most important modifications of flavonoids is the addition of variable sugar moieties (such as rutinose addition to hesperetin or quercetin, forming hesperidin and rutin, respectively), further increasing their structural diversity and function. This important group of phytochemicals gained interest due to its health‐promoting properties, including antitumor, antibacterial, antifungal and also antiviral actions (Panche *et al*., 2016). During the last decade, multiple biotechnological efforts have resulted in the production of dozens of flavonoids. The combinatorial nature of flavonoids biosynthesis together with cutting‐edge synthetic biology approaches allows the possibility to (i) synthetize novel flavonoids with widespread and still unexplored applications and (ii) screen these new chemical structures towards added‐value pharmacological applications, such as antiviral drugs. Microbial biotechnology allows the possibility to implement these two steps in the same bioprocess, thus providing an optimal framework for this fast repositioning.

Since the outbreak of the first severe acute respiratory syndrome (SARS) in 2002/2003, caused by SARS‐CoV, the outbreak of MERS in 2012 (caused by MERS‐CoV) and the COVID‐19 pandemic in 2019/2020 (caused by SARS‐CoV‐2), different antivirals and natural compounds have been tested against coronaviruses, such as remdesivir, ribavirin or herbacetin (Cherian *et al*., 2020). Of the natural anti‐coronaviral compounds, flavonoids in particular have shown interesting inhibitory bioactivities (Russo *et al*., 2020) that will be described in detail. In this review, we first give an overview of studies observing potential antiviral properties of this family of natural bioactive compounds against human (+)ssRNA viruses (such as coronaviruses and others displaying identical replication cycles). Here, we further summarize computational studies targeted on the identification of key chemical signatures involved in these antiviral properties. Finally, we show the way forward for updating a classical industrial biotechnology DBTL cycle towards the rational design, construction and screening of combinatorial libraries of flavonoids with antiviral properties.

## Antiviral properties of flavonoids against coronaviruses

### Flavonoids inhibiting entry into host cell

**Fig. 1:**
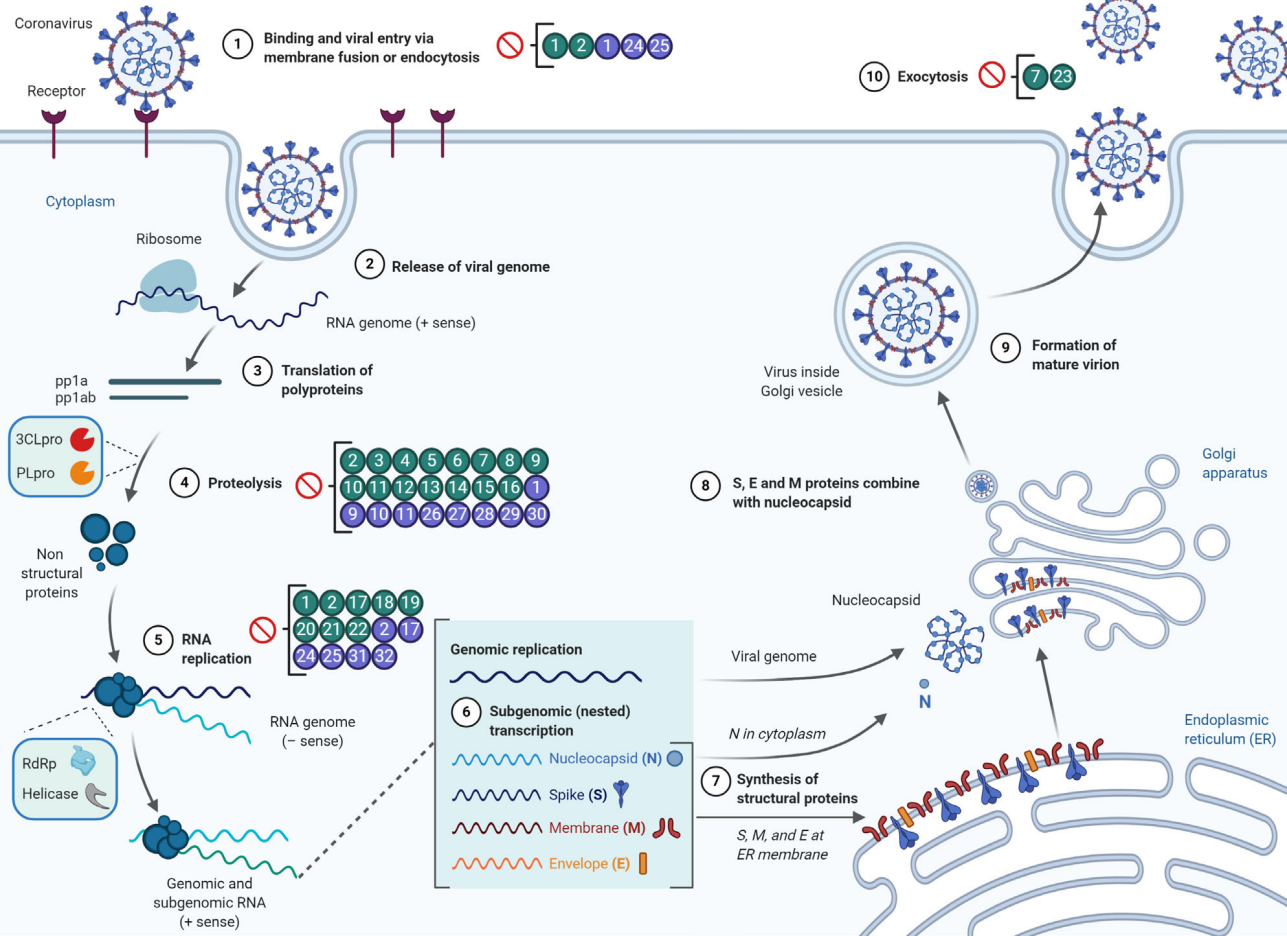
Overview of the coronavirus life cycle, indicating the attachment to the host cell membrane receptor, the translation of the viral (+)ssRNA genome in both polyproteins, the proteolysis carried out by 3CL^pro^ and PL^pro^ proteases, the viral genome replication steps and the virion maturation along endoplasmic reticulum and Golgi apparatus, with final exocytosis across the cell membrane. Numbers encircled in green represent flavonoids tested *in vivo* against SARS‐CoV and/or MERS‐CoV. Numbers encircled in purple represent flavonoids identified *in silico* as promising drugs against SARS‐CoV‐2. Numbers have been assigned as follows: **1**: luteolin; **2**: quercetin; **3**: tomentin B; **4**: isobavachalcone; **5**: 4´‐O‐methylbavachalcone; **6**: papyriflavonol A; **7**: kaempferol; **8**: quercetin‐3‐β‐galactoside; **9**: hesperetin; **10**: amentoflavone; **11**: GCG; **12**: herbacetin; **13**: rhoifolin; **14**: pectolinarin; **15**: quercetin‐3‐β‐D‐glucoside; **16**: helichrysetin; **17**: myricetin; **18**: scutellarein; **19**: baicalein; **20**: silibinin; **21**: quercetagetin; **22**: luteolin‐7‐d‐glucoside; **23**: juglanin; **24**: hesperidin; **25**: EGCG; **26**: glabrene; **27**: glabrone; **28**: isosilybin A; **29**: robustone; **30**: cinnamtannin; **31**: baicalin; **32**: diosmin. *Created with BioRender.com*.

SARS‐CoV and SARS‐CoV‐2 coronaviruses enter the human host cell after binding to the angiotensin‐converting enzyme 2 (ACE2) receptor at the cellular membrane (Fig. 1). This binding is established through the viral spike glycoprotein (at its S1 domain), which protrudes from the virus envelope. Once attached, the S2 domain (fusion peptide domain, identical protein sequences in SARS‐CoV and SARS‐CoV‐2) is necessary for the fusion of viral and cellular membranes and the virus endocytosis (Coutard *et al*., 2020). This process requires the protease activation of the spike glycoprotein at a furin site placed between the S1 and S2 domains. The expression of ACE2 membrane protein receptor in different cell types, such as in the airways and lungs, makes these tissues susceptible to virus infection (De Clercq, 2006; Adedeji *et al*., 2013). The spike glycoproteins of SARS‐CoV and SARS‐CoV‐2 share 76% amino acid sequence identity (Fig. S1; Wu *et al*., 2020). The flavone luteolin inhibited the attachment and entry of SARS‐CoV virions into human Vero E6 cells, probably after binding to the S2 domain of the spike glycoprotein, showing an EC_50_ of 10.6 µM, without toxicity to human cells (CC_50_ of 0.155 mM via MTT cell viability assay; intraperitoneal LD_50_ in mice was 232.2 mg kg^−1^ of body weight) (Yi *et al*., 2004). In the same inhibition assay, the flavonol quercetin showed an EC_50_ of 83.4 µM (Yi *et al*., 2004).

### Flavonoids inhibiting polyprotein maturation

In coronaviruses, the production of the replication–transcription complex is initiated by the translation of genes ORF1a and ORF1b, which encode two large polyproteins (pp1a and pp1ab). Two viral proteases process both polyproteins, 3CL^pro^ (3‐chymotrypsin‐like protease, also called main protease, M^pro^) and PL^pro^ (papain‐like protease), a cysteine protease (Fig. S1) (Ziebuhr *et al*., 2001; Thiel *et al*., 2003; Ziebuhr, 2004). This is a key step during viral multiplication and makes these proteases attractive targets for antiviral drugs.

For example, in a cell‐free cleavage and a cell‐based protease cleavage assay, hesperetin was the most potent inhibitor of SARS‐CoV 3CL^pro^ with an IC_50_ of 8.3 µM (Lin *et al*., 2005), while daidzein showed only activity in the cell‐free, but not in the cell‐based assay. In another study, quercetin‐3‐β‐D‐galactoside showed an inhibitory activity with an IC_50_ of 42.79 µM (Chen *et al*., 2006). The molecular‐modelling‐driven biosynthesis of eight new quercetin‐3‐β‐galactoside derivates and subsequent 3CL^pro^ inhibition studies revealed the importance of hydroxyl groups of the quercetin moiety in 3CL^pro^ inhibition, since their removal caused a substantial reduction in the inhibitory activity (Chen *et al*., 2006). Another study revealed the biflavone amentoflavone (IC_50_ of 8.3 µM) as one of the most potent 3CL^pro^ non‐competitive inhibitors. A comparison of amentoflavone with three other biflavones revealed that the methylation of 7‐,4ʹ‐ and 4ʹ″‐hydroxyl groups reduced the inhibitory activity. In addition, three monoflavones, apigenin, luteolin and quercetin, showed higher IC_50_ values of 280.8, 20.2 and 23.8 µM respectively. These data strengthen the assumption that hydroxyl groups, here at the C‐3´ position, such as in luteolin or quercetin and the larger structure of biflavones enhance inhibition activity against SARS‐CoV 3CL^pro^ (Ryu *et al*., 2010). Another important structural feature of flavonoids might be the galloyl group of gallocatechin gallate (GCG), which was suggested to contribute to the good inhibition values of GCG (IC_50_ of 47 µM) because this trihydroxylated aromatic moiety establishes four hydrogen bond interactions in the active site of 3CL^pro^ (Nguyen *et al*., 2012). A large study (Jo *et al*., 2020a) using 64 flavonoids revealed the flavonol herbacetin (IC_50_ of 33.17 µM) and the flavone glycosides rhoifolin (27.45 µM) and pectolinarin (37.78µM) as the most promising flavonoids. A docking simulation revealed the 8‐hydroxyl group in herbacetin as critical for its high binding affinity, and likely, the sugar moieties of rhoifolin and pectolinarin also contribute to the high binding affinity to SARS‐CoV 3CL^pro^ (Jo *et al*., 2020a). Herbacetin was also one of the most promising inhibitors of MERS‐CoV 3CL^pro^ with an IC_50_ of 40.59 µM (Jo *et al*., 2019). Similarly, effective were quercetin 3‐β‐D‐glucoside and the two chalcones: isobavachalcone and helichrysetin with IC_50_ values of 37.03, 35.85 and 67.04 µM respectively. In the case of the two chalcones, helichrysetin and isobavachalcone, the flexibility of their chalcone scaffold was suggested to lead to a more effective binding to MERS‐CoV 3CL^pro^ (Jo *et al*., 2019). These results are interesting, since most of the amino acids involved in binding of flavonoids to MERS‐CoV 3CL^pro^ are conserved (Fig. S1).

All these studies demonstrate that many flavonoids are effective compounds to inhibit coronaviral 3CL^pro^. Since SARS‐CoV‐2 3CL^pro^ shares 96% amino acid sequence identity with SARS‐CoV 3CL^pro^ and most of the amino acids involved in binding with MERS‐CoV 3CL^pro^ (Chen *et al*., 2020), all this information is useful for the screening of available flavonoids and the development of new ones against SARS‐CoV‐2 3CL^pro^.

Three studies on PL^pro^ reported a number of flavonoids inhibiting this protease. Notably, several novel geranylated flavonoids with a 3,4‐dihydro‐2H‐pyran moiety were highly effective against SARS‐CoV PL^pro^, in particular tomentin B (with an IC_50_ of 5 µM, Cho *et al*., 2013). The chalcones isobavachalcone and 4ʹ‐O‐methylbavachalcone exhibited an IC_50_ in the range of 7.3–10.1 µM, which was much more bioactive than flavanones and isoflavones tested in this study (Kim *et al*., 2014). One of the lowest IC_50_ values against SARS‐CoV PL^pro^ was observed for papyriflavonol A, a prenylated quercetin derivate from *Broussonetia papyrifera* (IC_50_ of 3.7 µM). Its higher activity compared with flavones such as kaempferol (IC_50_ of 16.3 µM) or quercetin (IC_50_ of 8.6 µM) might be due to the prenyl group within the resorcinol moiety (ring B), which can form strong hydrophobic interactions with the protease. Also, the higher number of hydroxyl groups in quercetin compared with kaempferol seems to have a positive impact on the inhibitory activity (Park *et al*., 2017). While similarities between different coronaviruses are less than for 3CL^pro^, SARS‐CoV’s PL^pro^ still shows a very high amino acid sequence identity of 82.86%, making these studies a good starting point for screening and testing compounds against SARS‐CoV‐2 PL^pro^ as well (Fig. S2).

### Flavonoids inhibiting viral replication

The two large polyproteins pp1a and pp1b encode 16 non‐structural proteins (nsp1‐16) which are part of the viral replicase–transcriptase complex. Two important proteins in this replicase–transcriptase complex are the RNA‐dependent RNA polymerase (RdRP or nsp12) and the helicase (nsp13). Therefore, they can be feasible targets for the development of antivirals. Similar to the proteases, the helicase is a highly conserved protein in coronaviruses (Fig. S3), and therefore, flavonoids able to inhibit other coronavirus helicases can be also useful to target the recently discovered SARS‐CoV‐2 (Keum and Jeong, 2012; Yu *et al*., 2012; Mirza and Froeyen, 2020). Two *in vitro* FRET and colorimetry‐based ATP hydrolysis assays showed that the flavonoids myricetin, scutellarein and baicalein have a strong inhibitory activity (more than 90% at 10 µM concentration) of the ATP hydrolysis, but not of the RNA‐unwinding activity of the SARS‐CoV helicase. The IC_50_ values of myricetin, scutellarein and baicalein were 2.71 µM, 0.86 µM and 0.47 µM respectively. Neither myricetin or scutellarein exhibited cytotoxicity against human cells (Keum and Jeong, 2012; Yu *et al*., 2012; Keum *et al*., 2013). *In silico* modelling showed that these two flavonoids interact directly with the ATP/ADP binding pocket of the SARS‐CoV helicase (Keum and Jeong, 2012; Yu *et al*., 2012; Keum *et al*., 2013).

### Flavonoids inhibiting virions maturation and exocytosis

The ORF3a gene of SARS‐CoV encodes a membrane ion channel (3a channel) which is involved in the viral release process from the host cell. The activity of this channel causes a membrane depolarization and the activation of Ca^2+^ channels, which allows the virus release by exocytosis. This 3a ion channel is inhibited by the flavonoid kaempferol and by its glycoside derivatives, such as juglanin (IC_50_ of 2.3 µM), which have a stronger inhibitory effect than kaempferol (Schwarz *et al*., 2014). The sequence identity between the 3a ion channel in SARS‐CoV and SARS‐CoV‐2 is 73% (Fig. S4), which may indicate that kaempferol may also inhibit the channel in the novel coronavirus.

A host enzyme which is necessary for viral maturation of SARS‐CoV‐2 is furin, a cellular proprotein convertase which cleaves the spike protein between the S1 and S2 sites and is responsible for infection of human lung cells (Hoffmann *et al*., 2020). This cleavage site in the spike protein is absent in SARS‐CoV and MERS‐CoV (Coutard *et al*., 2020), but found in several other viruses, such as dengue (Yu *et al*., 2008). Luteolin was observed to inhibit the maturation of dengue virions through the inhibition of furin (Peng *et al*., 2017), a mechanism which may be of interest also in coronavirus maturation.

### Experimental evidence of flavonoids inhibiting a SARS‐CoV‐2 enzyme

During the revision of this manuscript the, to our knowledge, first experimental evidences of flavonoids inhibiting 3CL^pro^ of SARS‐CoV‐2 were published (Abian *et al*., 2020; Jo *et al*., 2020b). In one study, 70 flavonoids were tested for their inhibition on 3CL^pro^ activity (Jo *et al*., 2020b). Especially baicalin, a glucuronated flavone showed an effective inhibition (IC_50_ of ~ 35 µM). Two rutinosylated flavonoids, the flavone pectolinarin and the flavonol herbacetin also proved to prominently inhibit 3CL^pro^ (IC_50_ of ~ 54 and 51.5 µM respectively). Interestingly, baicalin showed only low inhibition on the catalytically active peptide from SARS‐CoV 3CL^pro^ in a previous study, in which rhoifolin showed higher inhibitory effects on SARS‐CoV 3CL^pro^ (Jo *et al*., 2020a). This suggests that despite a 96% amino acid sequence identity, the binding of inhibitors can be severely different, even though the fact that only the active site peptide was used from SARS‐CoV 3CL^pro^ might contribute to that difference. Corresponding docking studies suggest that the RutinOSylated compounds bind differently than baicalin (Jo *et al*., 2020b).

The second study demonstrated an inhibitory activity (IC_50_ of 21 µM) of quercetin against SARS‐CoV‐2 3CL^pro^ (Abian *et al*., 2020). This is contrary to the before‐mentioned study by Jo et al., in which quercetin did not show an inhibitory effect on 3CL^pro^ at a concentration of 80 µM. While the same methods were used to study inhibition (protease FRET assay), subtle differences as such in substrate concentration (20 µM in Abian *et al*., 2020, 2.5 µM in Jo *et al*., 2020b) and temperature (310 K in Jo *et al*., 2020b, unknown in Abian *et al*., 2020) or pH (8.0 in Abian *et al*., 2020, 7.5 in Jo *et al*., 2020b) might lead to differences in inhibition. Abian and colleagues suggested that quercetin altered the thermal stability of SARS‐CoV‐2 3CL^pro^ causing a destabilization with a concentration‐dependent effect (Abian *et al*., 2020). While these initial results are promising and further flavonoids might prove effective also to other SARS‐CoV‐2 proteins, the comparison of these two studies shows how easily a slightly different experimental set‐up might change inhibition values, which makes cross‐study comparisons difficult.

## Computational‐based identification of key flavonoids chemical signatures responsible for antiviral activities

Computational‐based drug discovery is a rapid drug screening technology that can quickly screen compound libraries by calculating the Gibbs free energy between small molecule ligands and drug targets, such as viral enzymes. Better inhibition is usually reflected by more negative binding values. Due to the significant improvement of computer performance and the evolution of molecular dynamics algorithms, the screening of compound libraries that contain even billions of molecular structures can be realized in a relatively short time. The structural elucidation of the main SARS‐CoV‐2 proteins including 3CL^pro^ (Jin *et al*., 2020), the spike protein (Walls *et al*., 2020; Xia *et al*., 2020) and RdRp (Gao *et al*., 2020) paves the way for these molecular docking analyses. For easy handling, a docking server focused on SARS‐CoV‐2 was made available (Kong *et al*., 2020, https://ncov.schanglab.org.cn/). However, because the structures were only published recently, some molecular docking studies relied on homology modelling of the SARS‐CoV‐2 proteins based on those from SARS‐CoV and/or MERS‐CoV as templates, a good option due to the relatively high similarity of the corresponding amino acid sequences (Figs S1–S4). A systemic study includes homology modelling of all SARS‐CoV‐2 proteins and docking of a range of phytochemicals interacting with all of these proteins (Wu *et al*., 2020). The current results from computational screening with flavonoids against SARS‐CoV‐2 proteins are listed in SI2 and concluded in Table 1.

**Table 1:** Promising flavonoids against SARS‐CoV‐2.

Flavonoid	Molecule/site of action	Class	IC_50_ (µM, SARS‐CoV)
Quercetin	3CL^pro 30^, PL^pro 1,2^, Spike ^3^	Flavonol	83.4 (EC50, cell entry)^4^, 23.8 (3CL^pro^)^5^, 8.6 (PL^pro^)^6^
Rutin	3CL^pro 7,8^, RdRP ^29^	Flavonol (glycoside)	N.d.
Diosmin	3CL^pro 7,9,10^	Flavone (glycoside)	N.d.
Hesperidin	3CL^pro 9,12,13,14^, Spike/ACE‐2 ^8^	Flavanone (glycoside)	N.d.
Epigallocatechin gallate	3CL^pro 10^, Spike^13^	Flavan‐3‐ol (gallate)	N.d.
Liquiritin	3CL^pro 15, 16, 30^	Flavanone (glycoside)	N.d.
Naringenin	3CL^pro 14^, human two‐pore channel^17^	Flavanone	N.d.
Helichrysetin	3CL^pro^	Chalcone	67.04 (MERS‐CoV)^18^
Quercetin 3‐β‐D‐glucoside	3CL^pro^	Flavonol (glycoside)	37.03 (MERS‐CoV)^18^
Isobavachalcone	3CL^pro^ PL^pro^	Chalcone	35.85^,^(MERS‐CoV)^18^ 7.3^19^
Pectolinarin	3CL^pro^	Flavone (glycoside)	37.78^20^
Rhoifolin	3CL^pro^	Flavanone	27.45^20^
Herbacetin	3CL^pro^	Flavonol	40.59 (MERS‐CoV)^18^ 33.17^20^
Gallocatechin gallate	3CL^pro^	Flavan‐3‐ol (gallate)	47^21^
Amentoflavone	3CL^pro^	Flavone (biflavone)	8.3^5^
Luteolin	3CL^pro^ Spike	Flavone	10.6 (Spike)^4^ 20.2 (3CL^pro^)^5^
Hesperetin	3CL^pro^	Flavanone	8.3^22^
Quercetin‐3‐β‐D‐galactoside	3CL^pro^	Flavonol (glycoside)	42.79^23^
Tomentin B	PL^pro^	Geranylated flavonoid	6,1^24^
4ʹ‐*O*‐Methylbavachalcone	PL^pro^	Chalcone	10.1^19^
Papyriflavonol A	PL^pro^	Flavonol	3.7^6^
Kaempferol	3CL^pro 30^, PL^pro^	Flavonol	16.3^6^
Myricetin	Helicase	Flavonol	2.71^26,27,28^
Scutellarein	Helicase	Flavone	0.86^26,27,28^
Baicalein	Helicase	Flavone	0.47^26,27,28^
Juglanin	3a channel	Flavonol (glycoside)	2.3^25^

These flavonoids exhibit a good score in computational docking studies and/or exhibited experimentally tested activities against SARS‐CoV or other coronaviruses. In addition, the listed flavonoids have good pharmacological applicability. Some of the flavonoids listed are rare and/or expensive to produce in an acceptable purity. Thus, efficient biotechnological production is a better alternative for these compounds.

^1^: Zhang *et al*. (2020a), ^2^: Sampangi‐Ramaiah *et al*. (2020), ^3^: Rane et al. (2020), ^4^: Yi *et al*. (2004), ^5^: Ryu *et al*. (2010), ^6^: Park *et al*. (2017), ^7^: Mittal *et al*. (2020), ^8^: Wu *et al*. (2020), ^9^: Chen *et al*. (2020), ^10^: Peterson (2020), ^11^: Bhowmik *et al*. (2020), ^12^: Joshi *et al*. (2020), ^13^: Tallei *et al*. (2020), ^14^: Utomo *et al*. (2020), ^15^: Zhu *et al*. (2020), ^16^: Zhang *et al*. (2020c), ^17^:Filippini *et al*. (2020), ^18^: Jo *et al*. (2019), ^19^: Kim *et al*. (2014), ^20^: Jo *et al*. (2020a), ^21^: Nguyen *et al*. (2012), ^22^: Lin *et al*. (2005), ^23^: Chen *et al*. (2006), ^24^: Cho *et al*. (2013), ^25^: Schwarz *et al*. (2014), ^26^: Keum and Jeong (2012), ^27^: Yu *et al*. (2012), ^28^: Keum *et al*. (2013), ^29^: Da Silva *et al*. (2020), ^30^: Vijayakumar *et al*. (2020). N.d, Not determined (docking studies only).

### Spike/ACE‐2 docking studies

Several docking studies investigating the interaction of flavonoids with the SARS‐CoV‐2 spike protein are available (Maiti and Banerjee, 2020; Yu *et al*., 2020; Tallei *et al*., 2020; for further preprints see SI2). Several studies also investigated the interaction of flavonoids with the human ACE‐2 receptor (Joshi *et al*., 2020; Bhowmik *et al*., 2020; Maiti and Banerjee, 2020; SI2). These interactions might also block the viral entry into host cells. After releasing the structure of the spike protein receptor binding domain (RBD) together with ACE‐2, the interaction of flavonoids with this structure has been elucidated as well (Utomo *et al*., 2020; Khan *et al*., 2020; Vijayakumar *et al*., 2020). Two flavonoids were observed to have very good binding values to either the spike protein or ACE‐2 in several studies, namely hesperidin (Joshi *et al*., 2020; Wu *et al*., 2020) and epigallocatechin gallate (EGCG) (Maiti and Banerjee, 2020; several preprints, see SI2). Quercetin, which inhibited the cell entry of SARS‐CoV, was observed to have a good binding value to spike in one study (Pandey *et al*., 2020); however, *in vitro* inhibition of SARS‐CoV 3CL^pro^ by quercetin was not observed (Lin *et al*., 2005). Luteolin, which inhibited cell entry of SARS‐CoV‐2 to a greater extent than quercetin, showed a binding value similar to quercetin (Pandey *et al*., 2020). Given the structural differences of SARS‐CoV spike and SARS‐CoV‐2 spike (Figs S1–S4, Lan *et al*., 2020), experimental studies should take a broad variety of flavonoids into account, with a focus on hesperidin, gallocatechin gallates and derivatives, since docking studies were promising across many studies.

### Protease docking studies

Most of the computational docking studies focused on SARS‐CoV‐2 3CL^pro^ as drug target (Chen *et al*., 2020; Fischer *et al*., 2020; Gao *et al*., 2020; Islam *et al*., 2020; Rasool *et al*., 2020; Vijayakumar *et al*., 2020; Da Silva *et al*., 2020; Zhang *et al*., 2020c, for preprints see SI2), also due to its central role in the viral life cycle (Fig. 1). Among those were extensive molecular docking studies with flavonoid libraries of up to 60 flavonoids potentially inhibiting 3CL^pro^ (Joshi *et al*., 2020; Rasool *et al*., 2020; Zhang *et al*., 2020c). While in general larger/bulkier flavonoids, such as gallates or glycosides, show better binding values, there is no consensus flavonoid showing good binding values across several studies. For example, luteolin was found to be the most promising flavonoid in one of the larger studies (Zhang *et al*., 2020c), but was not among the top hits in others (Rasool *et al*., 2020; Yu *et al*., 2020). Many promising flavonoids were only tested in a single study and need further investigation (e.g. glabrene and glabrone (Zhang *et al*., 2020c), isosilybin A and robustone (Rasool *et al*., 2020) or cinnamtannin (Joshi *et al*., 2020)). Hesperetin was observed to highly inhibit SERS‐CoV 3CL^pro^ (Lin *et al*., 2005) and showed a promising binding value (Utomo *et al*., 2020) even though its corresponding glycoside hesperidin showed a slightly better binding value and was also considered among the most promising flavonoids in other studies (Chen *et al*., 2020 and several preprints, see SI2). Bulkier flavonoids, such as the biflavone amentoflavone which showed good inhibition results with SARS‐CoV 3CL^pro^ *in vitro,* were found promising for *in silico* inhibition of SARS‐CoV‐2 3CL^pro^ (Mishra *et al*., 2020; Peterson, 2020). Equally, since galloyl groups might be important for inhibition of SARS‐CoV 3CL^pro^ (Nguyen *et al*., 2012), further investigation of catechin gallates as 3CL^pro^ inhibitors is justified.

### Docking studies with RdRp and other viral proteins

The RNA polymerase RdRP (Joshi *et al*., 2020; Lung *et al*., 2020; Vijayakumar *et al*., 2020; Da Silva *et al*., 2020) and other viral proteins (Wu *et al*., 2020; Yu *et al*., 2020; Zhang *et al*., 2020a) were investigated only in a few studies. However, several flavonoids putatively active against SARS‐CoV 3CL^pro^ or the spike protein were observed to have good binding values to one or more of the lesser studied drug targets as well. These flavonoids include baicalin, with good binding values to PL^pro^ and nsp9 (Wu *et al*., 2020) and hesperidin to nsp7_8 and the E‐channel (Wu *et al*., 2020), rutin to RdRP (Da Silva *et al*., 2020) plus some observations from preprints (SI2). In addition to the docking/molecular dynamics, two network analyses on SARS‐CoV‐2 drug finding were performed. One is based on COVID‐19 disease‐related genes and drugs targeting these genes, where also two flavonoids, myricetin and quercetin, were revealed to be promising drugs. The other study used unsupervised learning, a machine‐learning method, on gene expression profiles of SARS‐CoV‐2‐infected vs non‐infected cells (Taguchi and Turki, 2020), by which also quercetin was detected as a potential flavonoid drug against COVID‐19.

### Which flavonoids fit best? An outlook and a critical view on computational drug screening

In short, computational‐based drug discovery has led to the screening of many flavonoids potentially inhibiting SARS‐CoV‐2 proteins. These flavonoids cover the vast majority of flavonoid structures. Among them, flavones, flavanones and flavonols are the three classes with the most promising compounds (Table 1, Fig. 1). Due to the diversity of hydroxyl and methoxy modifications on the backbone, a number of different flavonoids can effectively fit into the binding pockets of protein targets. Besides, the addition of sugars can improve the hydrophilicity and hydrogen bonding capability of flavonoids and one must note that orally administered flavonoids are altered in the hepatic metabolism, products of which were found to have different binding capacities and could alter viral inhibition (Da Silva *et al*., 2020). Other modifications, such as the addition of fatty acids or gallate moieties on the backbone carbon atoms of flavonoids, also enhance structural diversity. This diversity is also much needed after possible mutations of viral proteins. However, due to the limits of algorithms and the complexity of protein inhibition, the results obtained from computational‐based screening might not always reflect the inhibition in biological systems accurately and requires validation with *in vitro* tests. In addition, drugs are not limited to target inhibition. Many potential medicines can activate the human immune system and suppress viral infection through the classic immunization route. For example, liquiritin was suggested to mimic type I interferon instead of inhibiting 3CL^pro^ (Zhu *et al*., 2020). Therefore, based on the prediction results *in silico*, the second stage should focus on molecular and cellular pharmacological screening. For this purpose, it is important to *synthetize* flavonoid compounds with structural diversity.

## From plants‐ to microbial community‐based biotechnological production and testing of flavonoids

Despite the broad application of flavonoids in human health, their industrial production is still dependent on plant‐based extraction. Unfortunately, the plant‐based production of these compounds at large scale is challenging as it faces long culture periods, specific cultivation requirements, heterogeneous mixtures and low abundance of molecules of interest in the source species (ranging between 100 and 1000 ppm, mg per kg plant) (Miean and Mohamed, 2001). Attempts to engineer native plants to accumulate target metabolites at higher levels have been addressed, but only with limited success because metabolic engineering is technically challenging in plants (Tatsis and O'Connor, 2016). One solution is chemical synthesis, but when dealing with metabolites with complex chemical structures, their production is extremely challenging due to multistep procedures and the required complex protecting group strategies (Ho *et al*., 2016). An attractive alternative is to express these plant biosynthetic pathways in microbial factories, using synthetic biology approaches. Many efforts in this sense during the last decade have resulted in the biotechnological production of dozens of flavonoids (Fowler and Koffas, 2009; Koirala *et al*., 2016; Pandey *et al*., 2016; Zha and Koffas, 2017). With very few exceptions, the achieved yields are very low (few mg/L), making hard their cost‐effective production (Pandey *et al*., 2016; Yang *et al*., 2020). This is mainly because the complexity of flavonoids’ biosynthetic pathways involves dozens of enzymatic steps and intricate regulatory networks. In response, metabolic engineering efforts have dramatically increased in complexity, turning from the modification of a small number of genes to complex designs involving dozens of genes with different metabolic functions. However, several obstacles arise when introducing high dosage of foreign proteins in a single bacteria including metabolic burdens (Wu *et al*., 2016b), unwanted interactions between genetic parts and overload of the cell capacity; all of these can result in decreased growth fitness and low production of the target metabolite (Johnston *et al*., 2020). In addition, the synthesis of complex natural metabolites often requires the synthesis of chemically unrelated intermediate precursors, thus hampering optimal flux distribution in the context of a single organism.

**Fig. 2:**
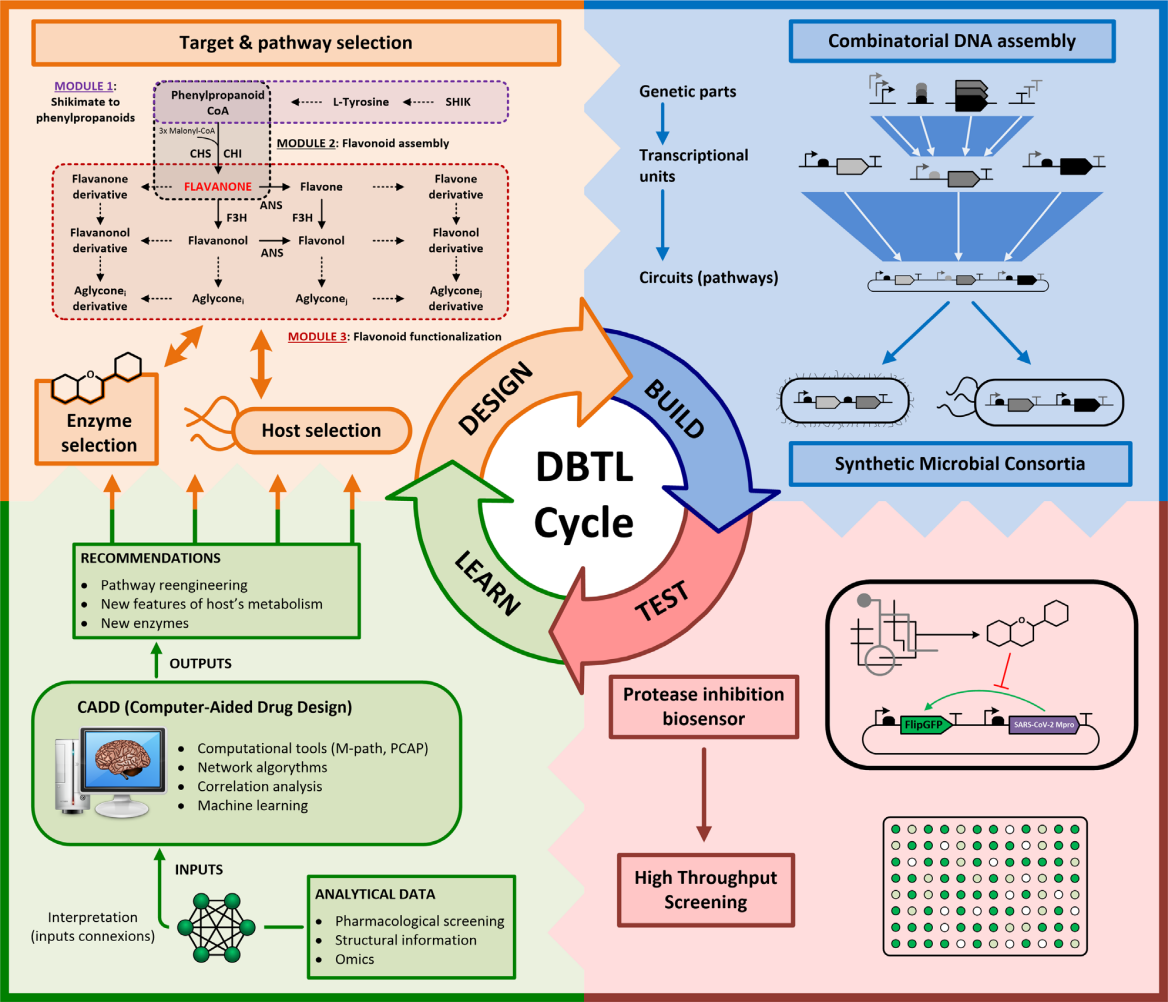
Iterative design of DBTL (*d*esign–*b*uild–*t*est–*l*earn) cycle for the identification of flavonoids as inhibitors of SARS‐CoV‐2 Mpro. Design step includes the selection of target compounds based on previous works, the *in silico* design of production pathways, optimal pathway segregation, identification of enzymes and the selection of microbial hosts to express the minimal metabolic modules (precursors, assembly and functionalization). Build stage is based on combinatorial DNA assembly methods to construct metabolic pathways that will be finally expressed in different components of a synthetic microbial consortium. Test stage includes production of the target compounds and the development of a synthetic protease inhibition biosensor to analyse biological activity using high‐throughput screening technologies. Learn stage processes the analytical data from the above steps and finds connections between compound structures, inhibition activities and metabolic fluxes to give recommendations to perform subsequent DBTL cycles.

The division of labour in the context of co‐cultures and/or microbial communities following the de‐coupling principle of synthetic biology (Endy, 2005) is emerging as highly promising approach to reduce this complexity while increasing yields. This approach relies on breaking biosynthetic pathways down to parts expressed in single organisms and to specifically tackle by separate, the challenges of flavonoid biosynthesis (Jones *et al*., 2016, 2017). Division of labour in different cells benefits metabolic processes in multiple ways: (i) by providing an expanded metabolic background, (ii) by avoiding competition by the same substrates/cofactors within a single cell, (iii) by allowing the appropriate combination of the different bioconversion pathways according to substrate composition, and (iv) by avoiding the important metabolic burdens imposed by the overexpression of multiple enzymes, and metabolite drainage for product synthesis in a single cell. In addition, community‐based biosynthesis also allows for real‐time monitoring of flavonoid biosynthesis (Xiu *et al*., 2017) and/or their potential effects (see Section Test step for a new envisioned biosensor). In flavonoid production, the application of this de‐coupling concept could manifest in the separation and optimization within the metabolic context of different strains of the minimal metabolic modules concurring in flavonoids biosynthesis including (i) the shikimate/phenylpropanoid pathway, producing precursors, e.g., phenylpropanoid motive, (ii) the flavonoid pathway, assembling central chalcones and (iii) the functionalization pathway, in which subgroups can be added (Fig. 2). For a current forum article on co‐culture for flavonoid synthesis, see Xu *et al*., 2020. Foundational examples of microbial communities used to produce flavonoids and other plant compounds include the use *of E. coli* polycultures for the production of anthocyanins (Jones *et al*., 2017), naringenin (Ganesan *et al*., 2017), flavan‐3‐ols (Jones *et al*., 2016), resveratrol (Camacho‐Zaragoza *et al*., 2016; Yuan *et al*., 2020), rosmarinic acid (Li *et al*., 2019) and pyranoanthocyanins (Akdemir *et al*., 2019). Recently, other non‐traditional microorganisms, such as *Yarrowia lipolytica,* have also been used for co‐culture‐based production of hydroxylated flavonoids (Lv *et al*., 2019). Overall, the effort done so far in this topic paves the way for development more integrative and automatized pipelines, such as microbial consortia fuelled DBTL cycles, towards the efficient biotechnological production of flavonoids.

## Tuning DBTL optimization cycles to the discovery of antiviral drugs

Design–build‐test–learn (DBTL) pipelines have been successfully applied in flavonoid production. Recently, the SYMBIOCHEM platform has addressed the production of the flavonoid pinocembrin through an automated DBTL pipeline targeted on pathway optimization. The pipeline computationally selected the most promising parts, e.g. enzyme coding regions and RBSs, and achieved up to 500‐fold improvements after two cycles (Carbonell *et al*., 2018). Despite this impressive improvement, the final titres (88 mg l^−1^ after 24 h) are still far from industrial cost efficiency which has been estimated to be at least 125 mg l^−1^ per h (Dr Jakob Ley, personal communication). Therefore, the biotechnological production of complex flavonoids remains challenging. Adding to the problem of biosynthetic limitations, current flavonoid‐based drug discovery screening and testing development rely on *in vitro* assays requiring pure or highly pure metabolites, thus adding complex and expensive purification steps to the flavonoid characterization process. Therefore, the search for new flavonoids with specific properties such as antiviral activities becomes an extremely time‐consuming task and the likelihood of achieving success is uncertain.

To deal with these limitations, the European consortium SynBio4Flav has recently proposed to bring together cutting‐edge DBTL cycles and division of labour in the context of multispecies consortia‐driven production of flavonoids (https://synbio4flav.eu/). SynBio4Flav addresses the reduction of complexity, strain‐specific metabolism refactoring and the overproduction of precursors, chemicals trafficking optimization and fermentation optimization. Here, we discuss a way to update the DBTL cycle concept to accelerate the synthesis and high‐throughput screening of new drugs (flavonoids) with antiviral properties against a selected target (SARS‐CoV‐2) in the context of microbial division of labour. Instead of discussing all the multiple drug targets in the coronavirus life cycle (see Sections Antiviral properties of flavonoids against coronaviruses and Computational‐based identification of key flavonoids chemical signatures responsible for antiviral activities, Table 1), we search exemplarily for inhibitors of 3CL^pro^. This enzyme has been identified as one of the principal targets for antiviral compounds and, as shown in Table 1, the number of flavonoids tested against this protease exceeds other targets and provides a good starting point to apply to a DBTL cycle. The cycle’s steps would include the following:

### Design step

This step defines the goal and selects the desired networks to be built, the necessary building blocks and the host organism(s) to be used based on previous knowledge. The huge structural diversity of flavonoids makes the systematic exploration of the full chemical space towards inhibition of SARS‐CoV‐2 3CL^pro^ certainly unapproachable. Thus, the design step should be profusely fed by previous experimental and computational chemical screenings as a qualified filtering step. Design should focus on basic structures displaying higher antiviral potential while providing the rational design of the target molecules. Flavanones, flavones, flavanonols and flavonols were found to be the most promising flavonoid aglycon structures against SARS‐CoV‐2 3CL^pro^ (Table 1). The production of flavanones through their precursors (phenylpropanoids‐CoA derivatives and malonyl‐CoA) is catalysed by the successive action of chalcone synthase, CHS and chalcone isomerase, CHI (Miyahisa *et al*., 2005) (Fig. 2). The subsequent identification of functionalization enzymes would allow the randomized and combinatorial modification of these core aglycones, resulting in a wide range of potential antiviral flavonoids. The envisioned biosynthetic pathway for flavonoid structures with potential activity against 3CL^pro^ is shown in Fig. 2. Selection of the enzymes responsible for these transformations is a key issue, as their activity strongly depends on their origin and on the substrate (Zhao *et al*., 2015; Malbert *et al*., 2018). Here, it can harness the potential of enzyme engineering and computational analysis to find and/or to engineer enzymes with high specificity and activity (Fehér *et al*., 2014; Carbonell and Trosset, 2015).

Selecting the appropriate host(s) in which the genetic machinery previously designed will be expressed is a critical step, especially in microbial consortia‐based biosynthetic approaches as that proposed in latest studies for microbial biosynthesis of flavonoids (Xu *et al*, 2020; Birchfield and McIntosh, 2020). In this context, prokaryotic (e.g. *E. coli*) and eukaryotic (e.g. *S. cerevisiae*) hosts with different, specialized metabolic properties can be used as successfully proven in synthesis of resveratrol (Yuan *et al*., 2020). The host‐module adaptation can be addressed in both directions: (i) the adaptation of the modules to the hosts will require an individualized selection of genetic parts, such as expression vectors, promoters and ribosome binding sites (Lee *et al*., 2011; Smanski *et al*., 2014; Jervis *et al*., 2019), and also, codon usage optimization (Tian *et al*., 2017); (ii) the adaptation of the host to the modules will require engineering some of the host’s features, such as optimizing metabolic fluxes, nutritional requirements and efficient secretion systems (Almaas *et al*., 2004; Mnif *et al*., 2012; Green and Mecsas, 2016). In addition to metabolic engineering, adaptive laboratory evolution has been profusely used to isolate more adapted and robust and reliable production strains (Williams *et al*., 2016; Calero and Nikel, 2019; Pereira *et al*., 2019; Vasconcellos *et al*., 2019). This host‐to‐module adaptation can be supported by computational algorithms that apply the increased understanding of microbial metabolism to produce tools that can predict phenotypic properties at genome‐scale level (Liu *et al*., 2015; Salguero *et al*., 2018; Choi *et al*., 2019).

### Build step

In this step, the synthesis and assembly of the different building blocks and their integration into the selected hosts are carried out. One of the central features of the flavonoid biosynthetic pathway is that it is non‐linear, thus resulting in a matrix‐based pathway in which the final product depends on the coordinates (Fig. 2). An increase in the matrix range by adding new reactions (rows and/or columns) will exponentially boost the number of potential products, with the possibility of synthetizing new‐to‐nature compounds with unexplored biological activities. Therefore, the ‘build’ step can largely benefit from DNA assembly tools that allow constructing genetic circuits in a sequential and combinatorial mode. In this sense, multipart (Gibson *et al*., 2009) and modular (Engler *et al*., 2009) DNA assembly tools are the most suitable methods for this purpose (Naseri and Koffas, 2020). These assembly methods can be applied to the construction of a flavonoid biosynthetic pathway. By using a unique set of building blocks, it is possible to construct a random collection of pathways to produce a large number of different flavonoids or to rationally construct a variety of pathways previously pre‐defined by the ‘design’ step. By combining these methods with high‐throughput screening techniques, it is possible to select optimal transcriptional regulators, promoters and terminators, as well as ribosome binding sites and other aspects that directly affect the enzymatic expression level (Naseri and Koffas, 2020).

Finally, the complex flavonoids biosynthetic pathway can be de‐convoluted into minimal functional modules and expressed in different and optimized hosts. This consortia‐based approach can help in simplifying the problem of pathway optimization, as each module can be individually optimized, and allows the à la carte combination to direct the production to target compounds. One of the principles of synthetic and systems biology is the integration of basic elements to create systems‐level circuitry (Purnick and Weiss, 2009). Synthetic microbial consortia apply this principle at cell level. In the context of consortia‐based flavonoid biosynthesis (Xu *et al*., 2020), it is possible to de‐convolute the complex biosynthetic pathway of flavonoids shown in Figure 2 into minimal functional modules (precursors, assembly and diversification). These modules can be expressed in different hosts and combined à la carte to increase metabolite diversity. This approach can also help in simplifying the problem of pathway optimization, as each minimal module can be individually developed before the final assembly (Jones *et al*., 2017). In addition, advances in the production of each of the functional modules, such as the drastically enhanced production of the precursor p‐coumaric acid in yeast (Liu *et al*., 2019), can be implemented in a faster way by exchanging the corresponding strains in the consortium.

### Test step

Here, all screening methods and biochemical analyses necessary to assess the effect of the integration of the engineered circuits into the host are implemented. In canonical DBTL cycles, testing steps are mainly conceived for the detection of clones harbouring correct biosynthetic pathways and/or optimized pathway performance, in terms of titres (Liu and Nielsen, 2019). For drug biosynthesis endeavours, testing steps acquire a dual functionality, e.g. first, the identification of clones synthetizing target metabolites and, subsequently, the identification of those with the optimal drug properties. In our study, the ‘test’ stage will be determined by the specific activity of flavonoids being synthetized as inhibitors of the SARS‐CoV‐2 3CL^pro^.

The inhibition of SARS‐CoV‐2 3CL^pro^ by different compounds has been widely studied using *in vitro* assays, where a fluorescent Dabcyl‐Edans substrate linked to the cleavage site of the protease is used as an inhibition reporter (Zhang *et al*., 2020b). These *in vitro* assays are quick and simple alternatives, and they are a good choice when pure compounds can be obtained as substrates.

DBTL cycles would allow to join chemical synthesis and antiviral testing in a single process, thus saving complex downstream and purification steps. For instance, the development of specific biosensors monitoring the inhibition of 3CL^pro^ by synthetized flavonoids will significantly accelerate the drug discovery process. This approach would allow drug biosynthesis and biological activity screening simultaneously in the same cell, thus overcoming additionally known limitations such as compounds toxicity, solubility and accessibility (Zha and Koffas, 2017). This protease inhibition biosensor could be developed using available technology, such as FlipGFP, a recently developed system based in the correct folding of a GFP‐based protein after the cleavage of a pre‐defined protease (Zhang *et al*., 2019). This reporter has been used to study apoptosis in animals (Zhang *et al*., 2019; Aoki *et al*., 2020; Jia *et al*., 2020) but its great potential has not been applied so far in bacterial biosensors. A FlipGFP edited with the 3CL^pro^ cleavage site and including the protease itself in one synthetic operon will be a novel biosensor to fulfil the objective of synthetizing and screening compounds at the same time (Fig. 2).

### Learn step

The data obtained in the above steps (Design, Build, Test) contain, among others, virtual and pharmacological drug screening data, analytical results for metabolic engineering, and enzymatic properties of enzymes involved in flavonoid biosynthesis. These data are interpreted in the Learn step to find connections between compound structures, inhibition activities and metabolic fluxes. The output of this step is a series of recommendations to drive subsequent DBTL cycles (Fig. 2). However, at present the Learn step is still the bottleneck in DBTL cycles. Analysing such large amounts of data systematically, automatically and intelligently, finding out the relationship between each factor and constructing reasonable mathematical models or/and networks to accurately predict reality, and finally applying the learning results to the next generation of optimizations remain great challenges.

Creating networks which graphically links the interrelationships of various factors with line connections and nodes has become the main method to intuitively reveal the relationship between each factor. A network based on drug repurposing platform can be established based on viral properties and drug characteristics (Li *et al*., 2020). For the metabolic engineering, a systemic characterization of genes related to biosynthetic metabolism according to their specific sequence/function signatures and metabolic regulators, followed by the construction of physical and genetic interaction networks, expose the metabolism’s regulatory network and enable access to an underexplored space in gene function (Mülleder *et al*., 2016). From the analysis of targeted intermediates, proteomics and metabolomics, several computational tools such as M‐path (Araki *et al*., 2015) and principal component analysis of proteomics (Alonso‐Gutierrez *et al*., 2015) make efficient use of chemical and enzymatic databases to find potential synthetic metabolic pathways or pinpoint specific enzymes, thus achieving higher production of target molecules from heterologous pathways. Similarly, a correlation analysis of targeted quantification of intermediates, proteins and metabolites in the relevant pathways can reduce pathway complexity and identify key proteins as the primary drivers of efficient product production, which benefits the construction and subsequently validation of conceptual models of pathway function (George *et al*., 2014).

In addition, machine learning has opened up the intelligence of data analysis and network construction on big data processing (Camacho *et al*., 2018). Drug discovery based on machine learning has also been widely and efficiently carried out with common drug screening models and algorithms. This further greatly improved the accuracy of predictions and sped up the prediction and decision in any link of the Learn step (Quest *et al*., 2010; Vamathevan *et al*., 2019). At present, due to the increasing amount of data, machine learning has entered the era of deep learning, which is a class of machine‐learning algorithms that uses artificial neural networks with many layers of non‐linear processing units for learning data representations (Chen *et al*., 2018). The rise of deep learning in each aspect of drug discovery has been reviewed recently (Zhang *et al*., 2017; Camacho *et al*., 2018; Chen *et al*., 2018; Ching *et al*., 2018). Meanwhile, deep learning has already extended their application in metabolic engineering and synthetic biology, especially in genomics, metabolic flux analysis and regulation design (Wu *et al*., 2016a; Camacho *et al*., 2018; Zou *et al*., 2019; de Jongh *et al*., 2020). Combination of these machine‐learning‐based methods can process biological experimental data more efficiently and could provide novel synthetic pathways and key enzymes for the biosynthesis of flavonoids. In addition, machine‐learning can enhance the directed evolution of enzymes via deep mutational scanning and design (Fox *et al*., 2007; Fowler and Fields, 2014; van der Meer *et al*., 2016; Starr and Thornton, 2017; Wrenbeck *et al*., 2017). A protocol of machine‐learning‐guided directed evolution for protein engineering introduces the steps required to build machine‐learning sequence‐function models, which can be an excellent method for the evolution of enzymes for flavonoid synthesis (Yang *et al*., 2019). Although machine learning still has disadvantages such as the requirement of large data samples and the lack of interpretability and repeatability, the application of machine learning can promote data‐driven decision making and also can reduce failure rates (Vamathevan *et al*., 2019). It is foreseeable that the learning step based on machine learning will connect theoretical and experimental data faster, smarter and more accurately, and ensures sped‐up drug/flavonoid design as an important response to fast‐evolving viruses.

## Outlook

Ongoing microbial biotechnology efforts can be reoriented in a straightforward manner for the fast treatment of emerging diseases such as COVID‐19. Among the myriad of compounds being currently tested against this disease, flavonoids emerge as promising antiviral agents targeting different life cycle steps of SARS‐CoV‐2. Despite the huge flavonoid chemical space available, only a small portion has been already screened, while a large amount remains unexplored. With the ever‐present threat of emerging pandemic coronaviruses, the proactive establishment of a chemical arsenal with potential activity against such viruses becomes critical. Microbial biotechnology, powered by new systems and synthetic biology tools, provides the optimal framework for the guided and targeted expansion of flavonoid chemical diversity. New approaches including the microbial community‐based biotechnological production of complex chemicals through a distributed catalysis and high‐throughput screening would largely accelerate this process.

## Conflict of interests

The authors declare no conflict of interests.

## Supporting information


**Fig. S1.** Sequence alignment of SARS‐CoV‐2 3CLpro (PDB accession number 6M2N), SARS‐CoV 3CLpro (PDB accession number 3TNS) and MERS‐CoV 3CLpro (PDB accession number 4WME) proteases. Two encircled amino‐acids (Hys41 and Cys145) constitute the catalytic dyad. Colored arrows indicate amino acids involved in the interaction between flavonoids and SARS‐CoV 3CLpro: Blue arrows show interactions with GCG; red arrows show interactions with amentoflavone; yellow arrow shows interactions with GCG and amentoflavone; green arrows show interactions with GCG, amentoflavone and quercetin‐3‐β‐D‐galactoside. Information of interactions was taken from (23–25). The method used for the alignment was ClustalW.
**Fig. S2.** Sequence alignment of SARS‐CoV‐2 PL^pro^ (GenBank accession number QHD43415.1) and SARS‐CoV PL^pro^ (PDB accession number 5TL6) proteases. The encircled amino‐acids (Cys114, Hys275 and Asp289) constitute the catalytic triad. The method used for the alignment was ClustalW.
**Fig. S3.** Sequence alignment of SARS‐CoV‐2 (GenBank accession number YP_009725308.1) and SARS‐CoV (Genbank accession number NP_828870.1) helicases. Only the amino‐acid at position 570 is different. The method used for the alignment was ClustalW.
**Fig. S4.** Sequence alignment of SARS‐CoV‐2 (GenBank accession number YP_009724391.1) and SARS‐CoV (Genbank accession number ABA02268.1) ion channel 3a, showing a 73% identity. The method used for the alignment was ClustalW.

**Table S1.** List of results from molecular in silico docking studies using flavonoids with SARS‐CoV‐2 (plus human ACE) proteins.
