# Supplementary material for: Repositioning microbial biotechnology against COVID‐19: the case of microbial production of flavonoids
Source: Microb Biotechnol. 2020 Oct 13;14(1):94–110. doi: 10.1111/1751-7915.13675 (PMC7675739; doi:10.1111/1751-7915.13675)
Supplement: Supplementary file 1 — Fig. S1. Sequence alignment of SARS‐CoV‐2 3CLpro (PDB accession number 6M2N), SARS‐CoV 3CLpro (PDB accession number 3TNS) and MERS‐CoV 3CLpro (PDB accession number 4WME) proteases. Two encircled amino‐acids (Hys41 and Cys145) constitute the catalytic dyad. Colored arrows indicate amino acids involved in the interaction between flavonoids and SARS‐CoV 3CLpro: Blue arrows show interactions with GCG; red arrows show interactions with amentoflavone; yellow arrow shows interactions with GCG and amentoflavone; green arrows show interactions with GCG, amentoflavone and quercetin‐3‐β‐D‐galactoside. Information of interactions was taken from (23–25). The method used for the alignment was ClustalW. Fig. S2. Sequence alignment of SARS‐CoV‐2 PLpro (GenBank accession number QHD43415.1) and SARS‐CoV PLpro (PDB accession number 5TL6) proteases. The encircled amino‐acids (Cys114, Hys275 and Asp289) constitute the catalytic triad. The method used for the alignment was ClustalW. Fig. S3. Sequence alignment of SARS‐CoV‐2 (GenBank accession number YP_009725308.1) and SARS‐CoV (Genbank accession number NP_828870.1) helicases. Only the amino‐acid at position 570 is different. The method used for the alignment was ClustalW. Fig. S4. Sequence alignment of SARS‐CoV‐2 (GenBank accession number YP_009724391.1) and SARS‐CoV (Genbank accession number ABA02268.1) ion channel 3a, showing a 73% identity. The method used for the alignment was ClustalW. [file MBT2-14-94-s001.docx]

**Supplementary figures**


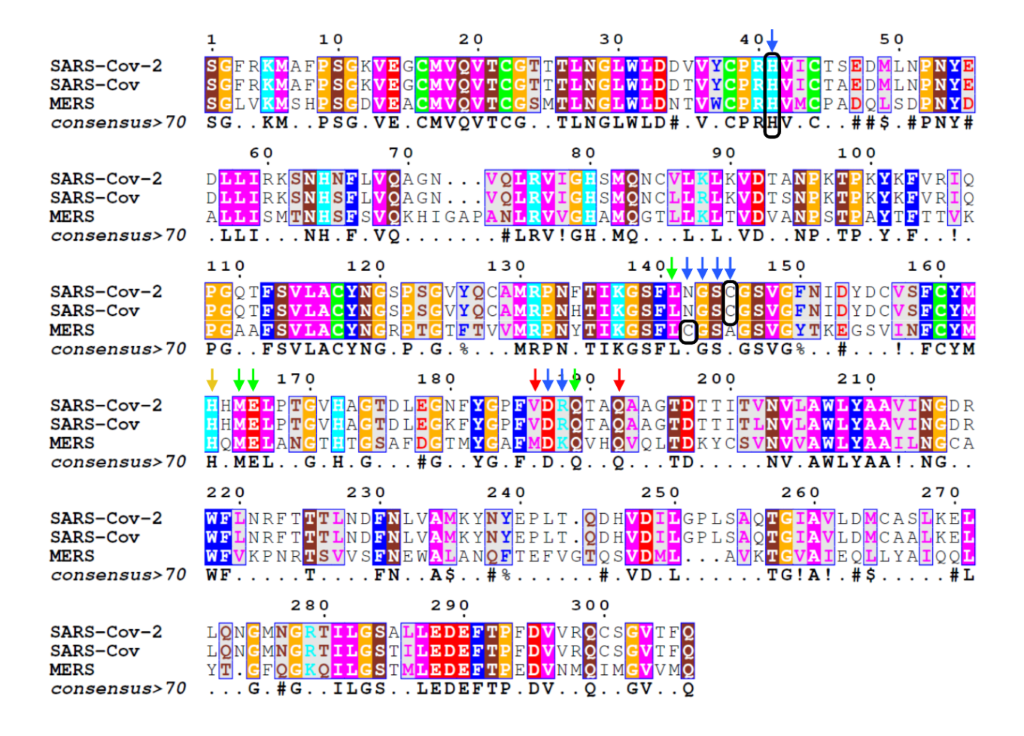


**Figure S1.** Sequence alignment of SARS-CoV-2 3CL^pro^ (PDB accession number 6M2N), SARS-CoV 3CL^pro^ (PDB accession number 3TNS) and MERS-CoV 3CL^pro^ (PDB accession number 4WME) proteases. Two encircled amino-acids (Hys41 and Cys145) constitute the catalytic dyad. Colored arrows indicate amino acids involved in the interaction between flavonoids and SARS-CoV 3CL^pro^: Blue arrows show interactions with GCG; red arrows show interactions with amentoflavone; yellow arrow shows interactions with GCG and amentoflavone; green arrows show interactions with GCG, amentoflavone and quercetin-3-β-D-galactoside. Information of interactions was taken from (23–25). The method used for the alignment was ClustalW.


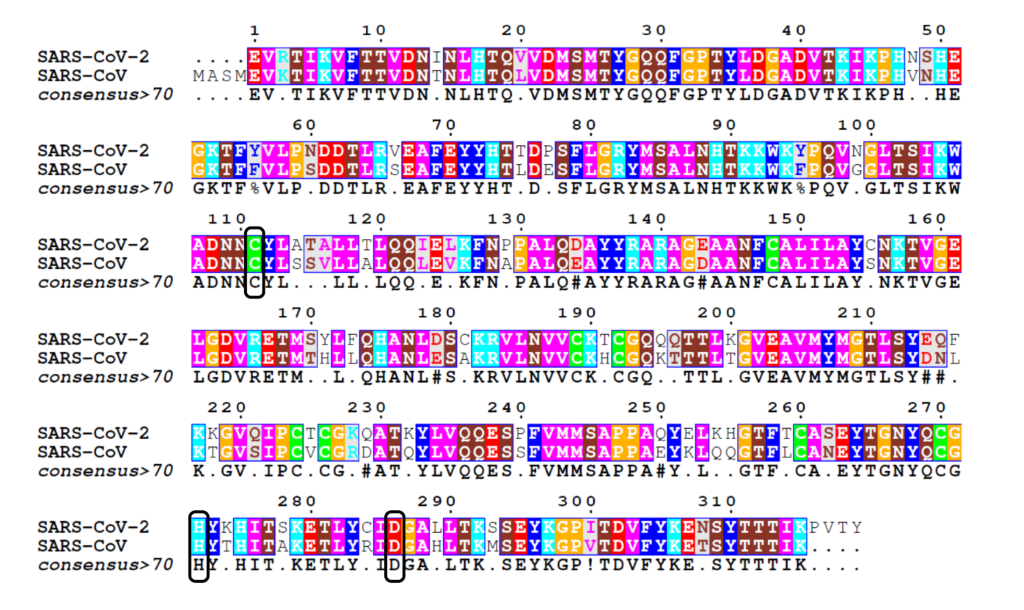


**Figure S2.** Sequence alignment of SARS-CoV-2 PL^pro^ (GenBank accession number QHD43415.1) and SARS-CoV PL^pro^ (PDB accession number 5TL6) proteases. The encircled amino-acids (Cys114, Hys275 and Asp289) constitute the catalytic triad. The method used for the alignment was ClustalW.


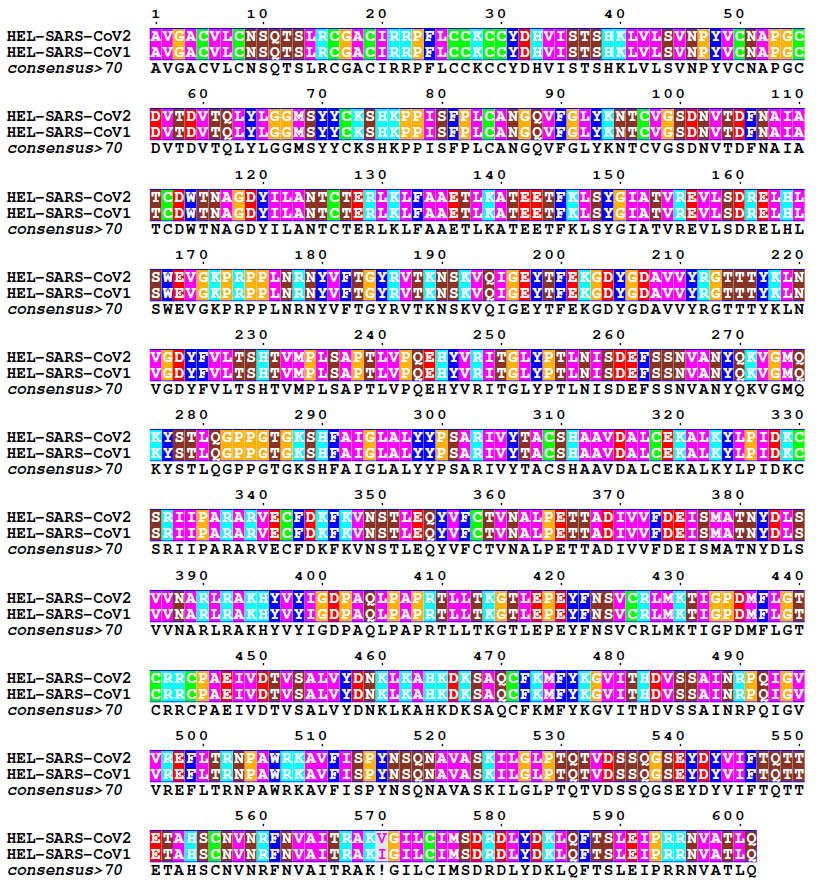


**Figure S3.** Sequence alignment of SARS-CoV-2 (GenBank accession number YP_009725308.1) and SARS-CoV (Genbank accession number NP_828870.1) helicases. Only the amino-acid at position 570 is different. The method used for the alignment was ClustalW.


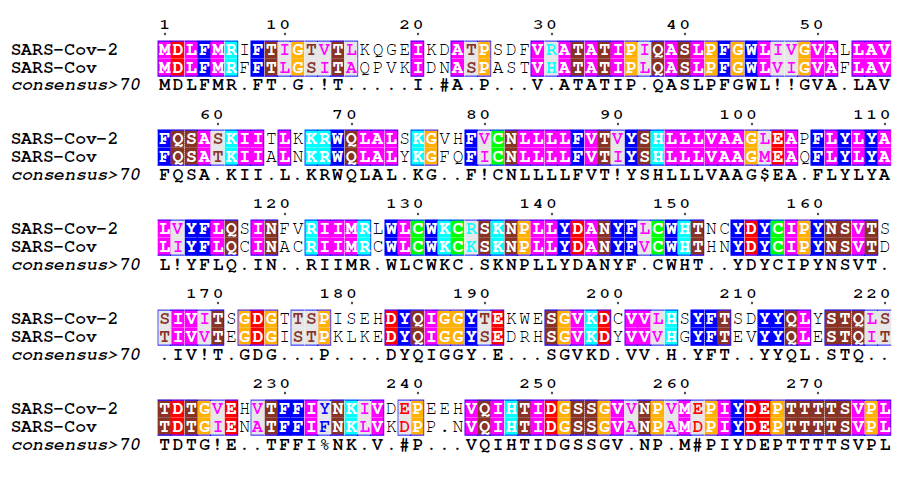


**Figure S4.** Sequence alignment of SARS-CoV-2 (GenBank accession number YP_009724391.1) and SARS-CoV (Genbank accession number ABA02268.1) ion channel 3a, showing a 73% identity. The method used for the alignment was ClustalW.
